# Editorial: Controlled environment agriculture goes dynamic

**DOI:** 10.3389/fpls.2026.1949519

**Published:** 2026-08-14

**Authors:** Kevin M. Folta, Ying Liu, Leo F. M. Marcelis, Elias Kaiser

**Affiliations:** 1 Horticultural Sciences Department, University of Florida, Gainesville, FL, United States; 2 East China Agri-Tech Center, Chinese Academy of Agricultural Sciences, Suzhou, China; 3 Horticulture and Product Physiology, Wageningen University, Wageningen, Netherlands; 4 Research Institute of Agriculture and Life Sciences, Seoul National University, Seoul, Republic of Korea

**Keywords:** controlled environment agriculture (CEA), crop management, dynamic optimization, fluctuating environment, greenhouse, vertical farm

## Introduction

In the first decade of the 21^st^ century Controlled Environment Agriculture (CEA) researchers, growers and enthusiasts started to push headlong into a seemingly new frontier in agriculture. Innovative LED-based arrays were emerging for commercial crop production and consumer hobbyist applications. They emitted an inviting pink glow, a blend of blue and red that appeared to be the fuel of a revolution. Vertical farms and some greenhouses were stacked with LED fixtures that promised to revolutionize CEA. However, initial efforts were sadly naïve efforts in underthinking.

It soon became apparent that static environmental conditions could not support profitable production. The most complex machine in these controlled environments was not the lights, climate control or hydroponics, but the plant itself. Strategy then shifted from fixed environmental parameters to follow the ever-changing development of the plant, opening opportunities for dynamic, data-driven crop management. By aligning fluctuating artificial lighting, changes in canopy structure, attention to physical root zones, and real-time plant physiology, growers and researchers aimed to reduce energy and labor costs while maintaining crop yields and quality (Kaiser et al., 2024).

This Research Topic focuses on a cutting edge honed by creative contemplation of plant biology and its interface with an artificial environment. The ten papers presented here focus on dynamic, variable lighting approaches, plant monitoring and modelling, rootzone and canopy management, and other innovative strategies to enhance plant growth and productivity in CEA.

## Optimizing dynamic lighting strategies

Artificial lighting is expensive, so Kong and Zhen examined whether dynamic lighting changes could save energy costs. Lettuce was grown under dark-light intervals of different lengths at the same daily light integral (DLI). Shorter intervals (20 min on/10 min off) reduced growth due to slow photosynthetic induction after light/dark transitions, while shorter intervals (10 min/5 min) had no negative effects.

Basil is a valuable herb whose growth depends on fluence rate and photoperiod. Akter et al. examined how increasing fluence rate and changing photoperiod affects production, compared to static conditions. Average DLI was identical between treatments. A dynamic change in fluence rate resulted in a 9% increase in dry weight and light use efficiency, while a dynamic change in photoperiod was the least efficient treatment.

Energy could also be saved by adjusting the spectrum. Blue light costs more to generate per photon than red, so Jadhav et al. tested how different combinations of blue could be mixed with red. Four treatments tested blue light reduction while keeping overall fluence rate constant. Reducing blue light by 25-38% increased fresh yield of baby leaf lettuce, due to increased water content.

The use of continuous lighting can be attractive especially in high-latitude greenhouse production, because of lower off-peak electricity costs. Lanoue et al. tested low-fluence rate continuous lighting on cherry tomatoes, only to find poor plant performance and yield. When the same DLI was split between white light days and low-fluence rate blue light at night, injury was mitigated, and fruit yield and quality increased.

End-of-production (EOP) lighting is an effective approach to improve product quality. Brewer et al. examined how halving fluence rate, combined with various blue/red ratios at EOP affects lettuce quality. Compared to untreated plants, Vitamin C in EOP treated plants increased significantly, while anthocyanin concentration and biomass decreased. Together, these studies demonstrate that short- or longer-term changes in fluence rate and/or light spectrum affect growth, yield and product quality.

## Root zone considerations, responsive covers, canopy management

Beyond light, structural adjustments to the canopy, root zone, and greenhouse materials affect crop performance. Langenfeld and Bugbee re-examined aeration effects in deep-flow hydroponics by distinguishing oxygen supply from agitation due to bubbling. Liquid culture with aeration (0–2 L min^–1^) was compared with peat-based soilless media that allows an undisturbed rhizosphere. Even gentle agitation disrupted rhizosphere function, impaired iron acquisition, and induced chlorosis. Hence, hydroponic management should consider the aeration-agitation trade-off, balancing dissolved oxygen supply with rhizosphere stability.

At the system-design level, Zamani et al. used smart covers to discuss how the greenhouse envelope could become a more responsive component of CEA. Their review highlights a transition towards adaptive greenhouse envelopes, particularly switchable covers, sensors and automated control systems.

Building on this plant-centered perspective, Kim and Kubota demonstrated how physiological thresholds can guide dynamic crop management. They developed a weekly light integral (WLI)-based pruning strategy in high-wire tomato, removing basal leaves only when WLI beneath the canopy was lower than the photosynthesis light compensation point of the lowest leaf. This approach reduced pruning events by 35-42% (potentially reducing labor costs) without reducing yield.

## The digital future: sensors, machine learning and digital twins

What if plants could control the light they received? Nam and Ferrarezi evaluated a control system that adjusted illumination based on real-time crop physiology. A new multiple linear regression model predicted the quantum yield of photosystem II using environmental variables, light history, and diurnal patterns. This machine learning (ML)-based approach compared sensor-based feedback control to constant lighting. While crop growth was similar across treatments, the sensor-based feedback system achieved the highest LED energy-use efficiency, demonstrating the viability of predictive ML in feedback-driven lighting control.

Frontzek et al. used an open-source Digital Twin framework to generate virtual replicas of actual farms. The study describes data management across diverse sensor and actuator networks using standardized interfaces. Modelica-developed models were exported as Functional Mock-up Units. A parameter estimation pipeline calibrates models against real-world data, while other sensors continuously track variables like biomass. The approach was validated through a hydroponic lettuce simulation, demonstrating high predictive accuracy and architectural feasibility.

## Conclusion

The gradual change from static ambient conditions to those that match the complexity of plant development likely has commercially important impacts. Studies presented here highlight a growing trend of detailed optimization, towards changing environmental variables to match plant needs. Future innovation will test how genotypic potential may be best tapped by an ever-fluctuating environment, monitored by sensors, and informed by plant-based feedback, ultimately lowering production costs and produce price and increasing grower profit.
